# Stem cell autocrine CXCL12/CXCR4 stimulates invasion and metastasis of esophageal cancer

**DOI:** 10.18632/oncotarget.15254

**Published:** 2017-02-10

**Authors:** Xingwei Wang, Yan Cao, Shirong Zhang, Zhihui Chen, Ling Fan, Xiaochun Shen, Shiwen Zhou, Dongfeng Chen

**Affiliations:** ^1^ Department of Gastroenterology, The Third Affiliated Hospital, Third Military Medical University, Chongqing 400042, China; ^2^ Department of Base for Drug Clinical Trial, The Second Affiliated Hospital, Third Military Medical University, Chongqing 400037, China

**Keywords:** esophageal cancer, esophageal cancer stem cells, CXCL12, CXCR4, metastasis

## Abstract

Esophageal cancer is one of the most common malignant tumors of the digestive tract. The greatest obstacle to the curing of esophageal cancer is its propensity to spread and metastasize. Esophageal cancer stem cells are considered the source for recurrence and metastasis of the tumors. While clinical evidence suggested that continuous up-regulation of CXCL12/CXCR4 was significantly associated with poor prognosis in patients with esophageal cancer, but the role and mechanism of CXCL12/CXCR4 in the invasion and metastasis of esophageal cancer has not been reported by far. This study found that esophageal cancer stem cells not only autocrine a great amount of CXCL12, but also high expression of its corresponding receptor CXCR4. Most importantly, the ability of esophageal cancer stem cells to spread and metastasize could be inhibited by blockage of CXCR4 with inhibitors or shRNA approaches both *in vivo* and *in vitro* studies. The important role of CXCL12 in the invasion and metastasis of esophageal cancer stem cells was also confirmed by loss-of-function and gain-of-function strategies. Mechanistically, we demonstrated that CXCL12/CXCR4 activated the ERK1/2 pathway and thereby ultimately maintained the characteristics of high-level invasion and metastasis of esophageal cancer stem cells. Taken together, our findings suggested that autocrine CXCL12/CXCR4 was one of the major mechanisms underlying the metastatic property of esophageal cancer stem cells through ERK1/2 signaling pathway, and might serve as a therapeutic target for esophageal cancer patients.

## INTRODUCTION

Esophageal cancer is one of the most common malignant tumors of the digestive tract. About 300,000 people died of esophageal cancer annually worldwide. Most patients with esophageal cancer were at the middle or advanced stage at the time of diagnosis when in most cases wide spread of cancer cells had already occurred, with a 5-year survival rate of only 15-25%. Tumor invasion and metastasis are the most important biological characteristics of malignant tumors which have significant impact on therapeutic effect and prognosis in patients with esophageal cancer [1–2]. Since Reya *et al*. came up with the theory of “tumor stem cells” in 2001, tumor stem cells have been considered to be responsible for recurrence and metastasis of tumors. Researchers have proven that tumor stem cells exist in various tumor tissues (breast cancer, colon cancer, ovarian cancer and lung cancer, etc.) and that this little group of tumor stem cells in tumor tissues plays an essential role in the recurrence and metastasis of tumors [3–4]. In addition, epithelial-mesenchymal transition (EMT) has been considered as the precondition of tumor cell invasion and metastasis. Recently, studies also showed that tumor cells transformed via EMT to gain the characteristics of tumor stem cells in order to reach the sites of metastasis where they proliferated subsequently to form new tumors, which provided new evidence for the important role of tumor stem cells in tumor invasion and metastasis [3–4].

Since 2003, Okumura et al. sorted surface markers with the low-affinity nerve growth factor receptor (or p75 neurotrophin receptor) and demonstrated that P75NTR-positive cells of normal esophageal epithelium were capable of proliferation, self-renewal and multidirectional differentiation and were identified as esophageal epithelial stem cells [5]. Subsequently, researchers sorted out esophageal cancer stem cells from esophageal cancer cells using the SP cell sorting approach with CD44, SOX2 and Lgr5 as the markers [6]. At the early stage, the applicant identified and cultured ECSCs using the SP cell isolation methods with OCT3 and SOX2 as the surface markers, and proved that the ECSCs cultured using both methods showed stronger disposition of invasion and metastasis compared with ordinary tumor cells [7], while the specific molecular mechanism was still unknown. By far, although the specific molecular mechanism by which ECSCs mediate the strong disposition of tumor metastasis through its extremely strong ability to invade and metastasize has been expounded in various tumor types, the molecular mechanism by which ECSCs involve in the invasion and metastasis of esophageal cancer has not been reported. Previous studies showed that the interaction between chemokines and their receptors plays an important role in the invasion and metastasis of tumor stem cells. For example, the pancreatic cancer stem cells CXCR4+/CD133+ have been considered an essential condition for the metastasis of pancreatic cancer [8]; breast cancer stem cells bind the CXCL12 ligand by high expression of CXCR4 and CCR7 on the cell surface, leading to metastasis of breast cancer stem cells [9]; ovarian cancer stem cells maintain strong disposition of invasion and metastasis through the binding of autocrine CCL5 to its own receptors, thereby plays an important role in the recurrence and metastasis of ovarian cancer [10]; breast cancer stem cells have also been proven to trigger the invasion and metastasis of breast cancer through CXCR1/CXCL8 [11]. Although the important role of chemokines and their receptors in the stem cells of a wide variety of tumors has been confirmed, the molecular mechanism by which they mediate the invasion and metastasis of ECSCs has not been reported. In this study, we found that autocrine CXCL12/CXCR4 was one of the major mechanisms underlying the metastatic property of ECSCs through ERK1/2 signaling pathway, and might serve as a therapeutic target in esophageal cancer patients.

## RESULTS

### Clinical samples suggested high expression of chemokine receptor CXCR4 in esophageal cancer stem cells

**Figure 1:**
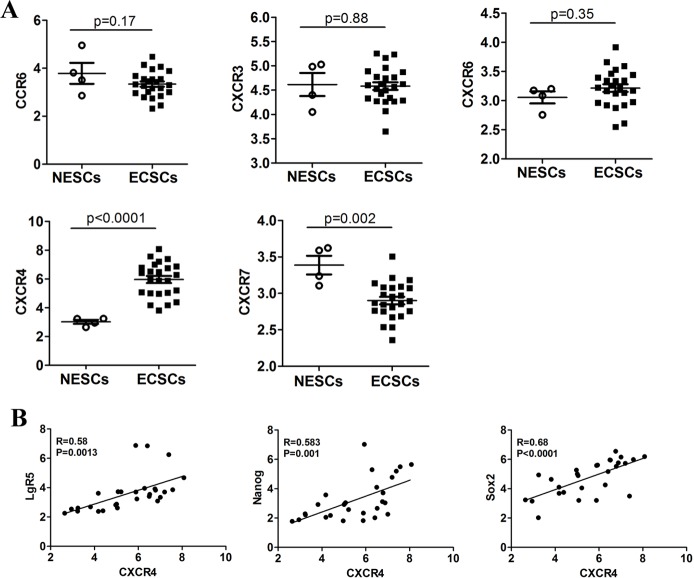
Expression of chemokine receptors in ECSCs **A**. Comparative analysis on the difference in the expression of chemokine receptors CCR6, CXCR3, CXCR6, CXCR4 and CXCR7 between normal esophageal stem cells (NESCs, n=4) and esophageal cancer stem cells (ECSCs, n=22). Statistically-significant differences were measured using an unpaired Student's t-test. **B**. The relationship between CXCR4 and stem cell markers (LgR5, Nanog and Sox2) expression was assessed using a Pearson correlation array (n=26).

Previous studies showed that the interaction between chemokines and their receptors plays an important role in the invasion and metastasis of tumor stem cells [8–11]. First, in order to identify the essential molecules mediating high level of invasion and metastasis of esophageal cancer stem cells, we retrieved data (NCBI GEO datasets: GSE65013) on expression of chemokine receptors in esophageal cancer stem cells (ECSCs) from 22 patients, and normal esophageal stem cells (NESCs) from 4 people. We compared the expression of chemokine receptors on the surface of ECSCs and NESCs, and found no significant difference in the expression of chemokine receptors CCR6, CXCR3 and CXCR6 between the two cell types (Figure 1A), while the expression of chemokine receptor CXCR4 was significantly higher in ECSCs than in NESCs (Figure 1A, p<0.0001), and the expression of chemokine receptor CXCR7 was significantly lower in ECSCs than in NESCs (Figure 1A, p=0.002). Lgr5, Nanog and Sox2 have been considered as genes of tumor stem cells, and researchers have proven the specific expression of these three genes in esophageal cancer stem cells. Therefore, we further analyzed the correlation between CXCR4 and the expression of these stem cell genes, and found that CXCR4 was positively correlated with stem cell genes Lgr5 (Figure 1B, R=0.58, p=0.0013), Nanog (Figure 1B, R=0.583, p=0.001) and Sox2 (Figure 1B, R=0.68, p<0.0001). The above results suggested high expression of CXCR4 in ECSCs and indicated that such high expression might contribute to the high propensity of invasion and metastasis of ECSCs.

### Autocrine secretion of CXCL12 and high expression of its corresponding receptor CXCR4 by ECSCs

**Figure 2:**
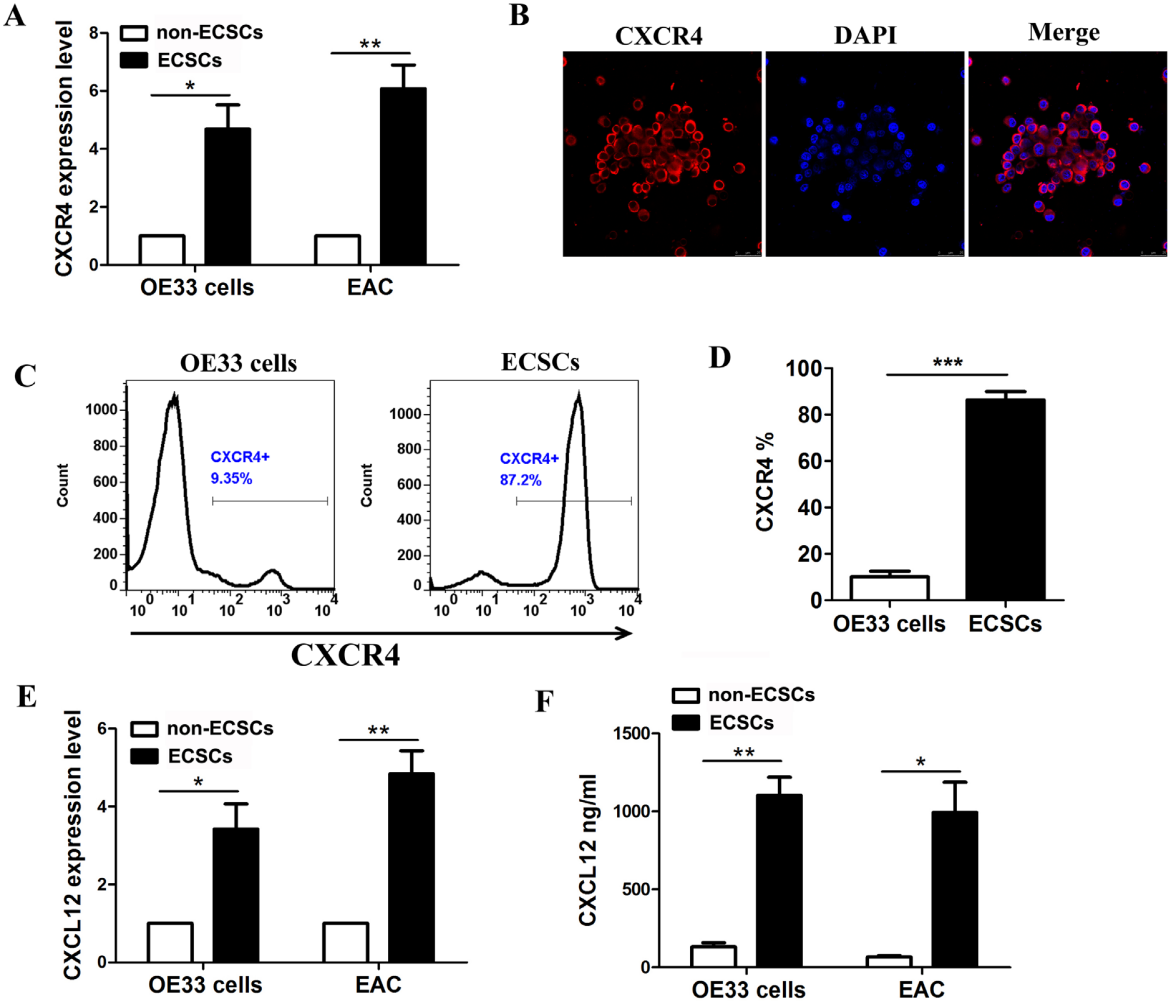
High expression of CXCR4 and autocrine secretion of CXCL12 by esophageal cancer stem cells **A**. Examination of CXCR4 expression on ECSCs from esophageal cancer cell line OE33 and esophageal cancer tissues by quantitative PCR test. **B**. Examination of CXCR4 expression on ECSCs by immunofluorescence (Magnification, 200×). **C-D**. Examination of CXCR4 expression on OE33 cells and corresponding ECSCs. **E-F**. Examination of CXCL12 secretion on ECSCs from OE33 and esophageal cancer tissues by PCR (E) and ELISA (F). Statistically-significant differences were determined using an unpaired Student's t-test, *p<0.05, **p<0.01, ***p<0.001.

As it is known to all, CXCL12/CXCR4 plays an important role in maintaining tumor invasion and metastasis [12–13]. We further sorted out ECSCs and non-ECSCs from OE33 cells and samples of esophageal cancer tissues by flow sorting [7], and determined the expression of CXCR4 by quantitative PCR. The results showed significantly high expression of CXCR4 in both OE33 cells derived or esophageal cancer tissues derived ECSCs (Figure 2A). Moreover, high expression of CXCR4 was seen at the cell membrane of ECSCs by immunofluorescence assay (Figure 2B). Flow cytometry confirmed that 87.2% of ECSCs from OE33 cells expressed CXCR4, while OE33 cells expressed 9.35% of CXCR4 (Figure 2C-2D). It is well known that chemokines exert their biological effects by binding to chemokine receptors. But do ECSCs also autocrinely secrete CXCL12? That is why we conducted the real-time PCR test to confirm that ECSCs, whether from OE33 cells or samples of esophageal cancer tissues, have significantly high expression of CXCL12 (Figure 2E); ELISA also confirmed the autocrine secretion of CXCL12 by ECSCs (Figure 2F). The above results sufficiently proved both high expression of CXCR4 and autocrine secretion of CXCL12 by ECSCs and suggested that CXCL12/CXCR4 might mediate the high propensity of invasion and metastasis of ECSCs.

### Significant inhibition of ECSCs’ ability to invade and metastasize after blockage of CXCR4

**Figure 3:**
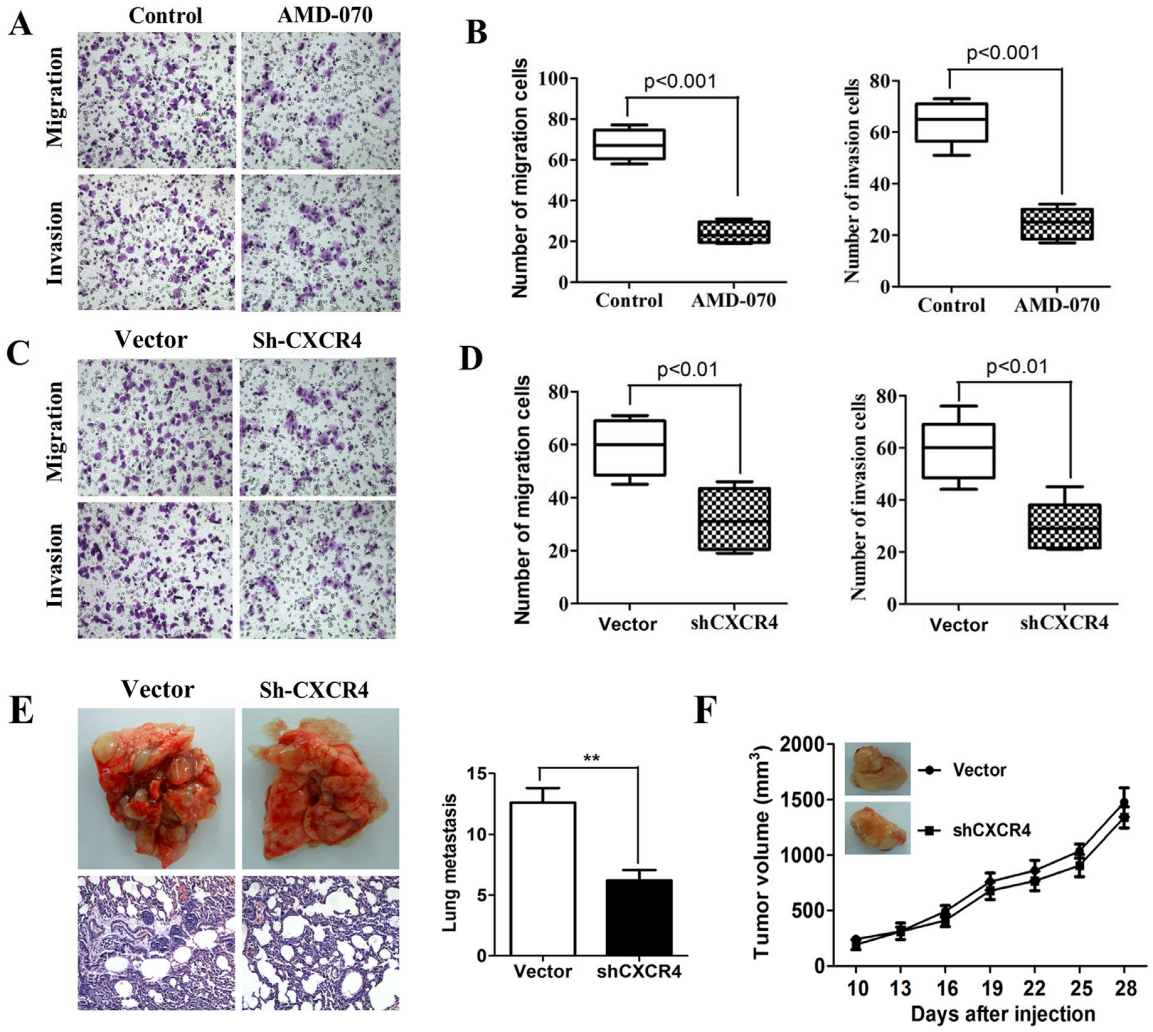
High propensity of invasion and metastasis of CXCR4 mediated ECSCs **A-B**. Adding CXCR4 inhibitor AMD-070 to ECSCs, incubating for 24 hours, and examining the change in ECSCs’ disposition of invasion and metastasis by transwell migration and matrigel invasion assays. **C-D**. Examination of the ability of vector and sh-CXCR4 infected ECSCs to invade and metastasize by transwell migration and matrigel invasion assays. **E**. 1×10^6^ vector or shCXCR4 transfected esophageal cancer stem cells were injected into the caudal vein, the mice were euthanized at 60 days, and status of pulmonary metastasis was observed. Pulmonary metastatic nodules were counted and HE staining was done. **F**. 1×10^6^ cells were implanted subcutaneously. The volumes of the tumors were measured every three days. At 50 days, the mice were euthanized. Statistically-significant differences were determined using an unpaired Student's t-test, **p<0.01.

Evidences including the important role of CXCL12/CXCR4 in tumor invasion and metastasis [12–13] and its strong correlation with the poor prognosis in patients with esophageal cancer [12–14] have been reported in literature. Therefore, in order to clarify the role of CXCL12/CXCR4 in the invasion and metastasis of ECSCs, we added a CXCR4 inhibitor AMD-070 to ECSCs, and observed its effect on ECSCs’ ability to invade and metastasize after incubation for 24 hours. Our transwell and matrigel invasion assays showed that after blockage of CXCR4, ECSCs’ ability to invade and metastasize was significantly inhibited (Figure 3A-3B, p<0.001). In addition, we transfected ECSCs with sh-CXCR4 and the control viral vector by the shRNA approach, and confirmed a transfection rate of above 85% by fluorescence microscope and FACS test (Supplementary Figure 1A-1B). The FACS test also proved significantly decreased expression of CXCR4 on the surface of ECSCs after transfection with shCXCR4 (Supplementary Figure 1C), and thereby proved our acquisition of ECSCs stably transfected with shCXCR4. Upon this basis, both transwell and matrigel invasion assays demonstrated that like treatment with CXCR4 inhibitor AMD-070, after transfection with shCXCR4, ECSCs’ ability to invade and metastasize significantly decreased (Figure 3C-3D, p<0.01). In order to further demonstrate the role of CXCR4 in the invasion and metastasis of ECSCs, we established a mouse caudal vein tumor xenograft model. 1×10^6^ vector or shCXCR4 transfected ECSCs were injected into the caudal vein. At 60 days, the mice were euthanized. Multiple and marked metastatic nodules were seen in the vector group, while significant reduction of pulmonary metastatic nodules was seen in the shCXCR4 transfection group (Figure 3E, p<0.01). This confirmed the important role of CXCR4 in the invasion and metastasis of ECSCs. In addition, in order to rule out the possibility that the significant reduction of metastatic tumors in the shCXCR4 group was caused by the impact of CXCR4 on the tumor formation ability of ECSCs, we established a mouse subcutaneous tumor transplantation model. 1×10^6^ vector or shCXCR4 transfected ECSCs were implanted in the left thighs of the mice. The volumes of the tumors were calculated every three days and showed no impact of CXCR4 on the tumor formation ability of ECSCs after transfection with sh-CXCR4 (Figure 3F, p<0.001). The above results showed that CXCR4 blockage can significantly inhibit ECSCs’ ability to invade and metastasize.

### CXCL12 is required for invasion and migration of ECSCs

**Figure 4:**
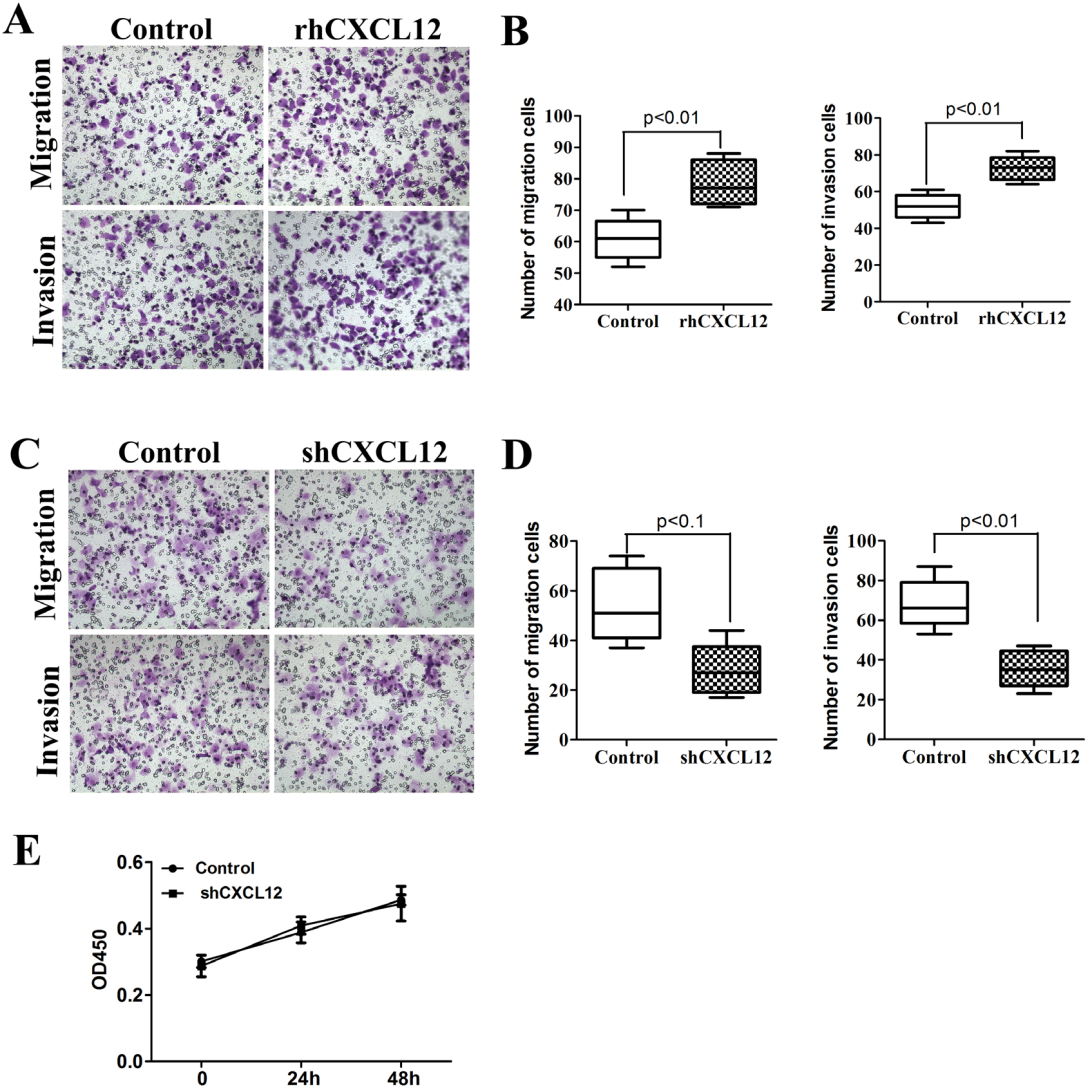
CXCL12 mediates ECSCs’ high propensity of invasion and metastasis **A-B**. ECSCs were added with rhCXCL12 and incubated for 24 hours. Transwell migration and matrigel invasion assays were carried out to examine ECSCs’ ability to invade and metastasize. **C-D**. Vector and shCXCL12 transfected ECSCs’ ability to invade and metastasize were examined by transwell migration and matrigel invasion assays. **E**. Examination of the proliferation of vector and shCXCL12 transfected ECSCs at 24 and 48 hours by the CCK-8 approach. Statistically significant differences were determined using an unpaired Student's t-test, and all tests were done in triplicates.

It was reported in previous literature that in the tumor microenvironment, CXCL12 secreted by stromal cells or immune cells had an effect on CXCR4 expressed by ECSCs, mediating its invasion and metastasis [12–13]. The results of our early-stage study proved that ECSCs were able to autocrinely secrete large amount of CXCL12. What role does CXCL12 play in the invasion and metastasis of ECSCs? Therefore, we added rhCXCL12 to ECSCs. After the transwell migration and matrigel invasion assays, we found that ECSCs’ ability to invade and metastasize was significantly enhanced after adding rhCXCL12 (Figure 4F, p<0.001). The results suggested that CXCL12 was able to enhance the invasion and metastasis of ECSCs. In order to further confirmed the important role of CXCL12 in the invasion and metastasis of ECSCs, we transfected ECSCs with sh-CXCR4 and the control viral vector by the shRNA approach, and confirmed a transfection rate of above 85% by fluorescence microscope and FACS test (Supplementary Figure 2A-2B). The ELISA test also proved significantly decreased secretion of CXCL12 on the surface of ECSCs after transfection with sh-CXCL12 (Supplementary Figure 2C), and thereby proved our acquisition of ECSCs stably transfected with sh-CXCL12. Most importantly, both transwell and matrigel invasion assays also found that after transfection with sh-CXCL12, ECSCs’ ability to invade and metastasize significantly decreased (Figure 4C-4D, p<0.01, p<0.001). In addition, we confirmed by using the CCK-8 test that no impact on ECSCs’ ability to proliferate occurred after transfection with sh-CXCL12 (Figure 4E). The above results sufficiently proved that the CXCL12/CXCR4 chemokine axis mediates the high propensity of invasion and metastasis of ECSCs.

### The CXCL12/CXCR4 chemokine axis is of vital importance to the activation of the ERK1/2 signal pathway on ECSCs

**Figure 5:**
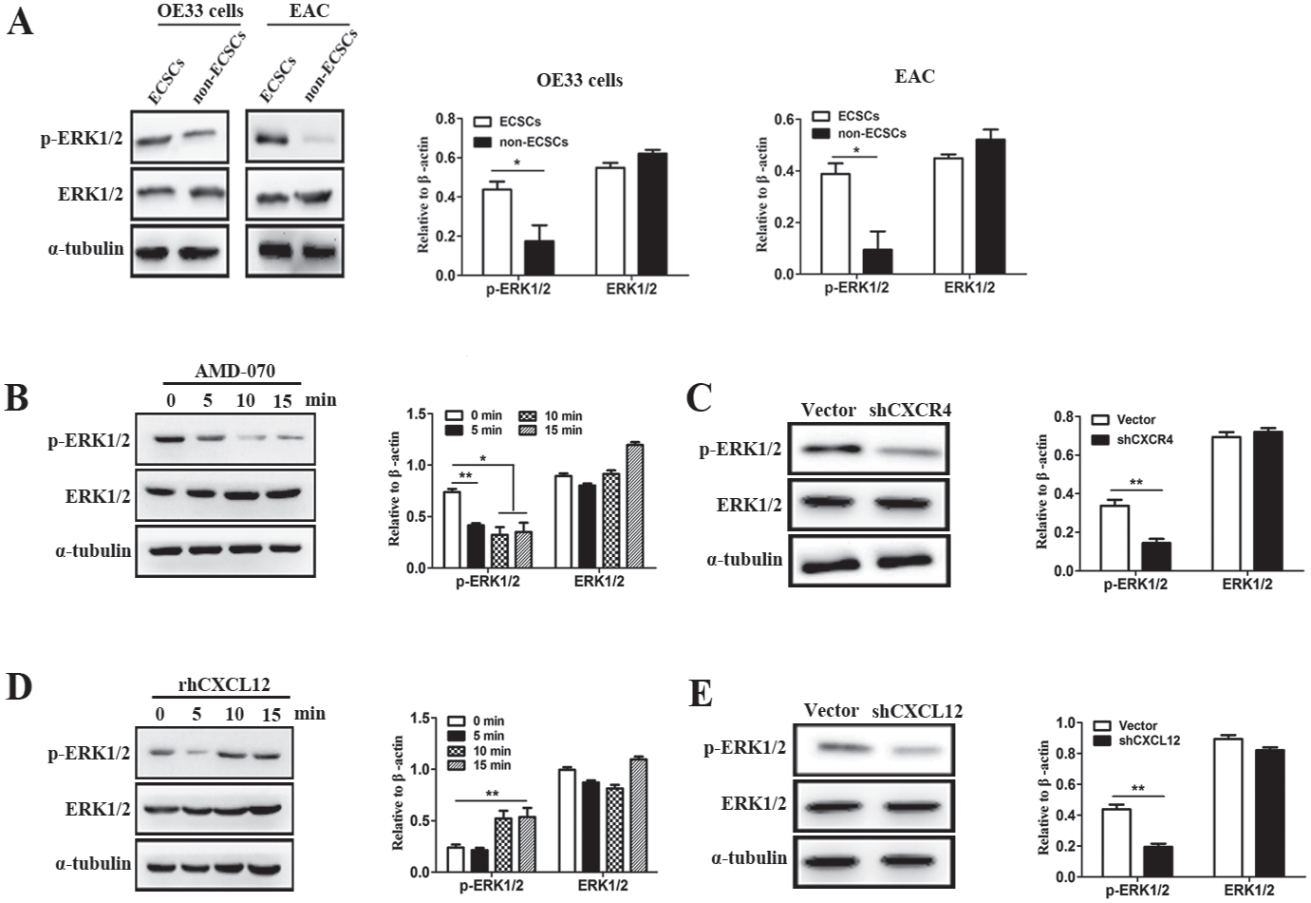
CXCL12-CXCR4 chemokine axis was of vital importance to the activation of ERK1/2 signal pathway on ECSCs **A**. Two cell groups, ECSCs and non-ECSCs, were selected by FACS from OE33 cells and tissues from patients with esophageal cancer. The expression of ERK1/2 and p-ERK1/2 were examined using western blot analysis (the results of statistical analysis were shown on the right). **B**. ECSCs from OE33 cells were added with AMD-070 and incubated for 0, 5, 10 and 15 minutes. The expression of ERK1/2 and p-ERK1/2 were examined using western blot analysis (the results of statistical analysis were shown on the right). **C**. The expression of ERK1/2 and p-ERK1/2 in vector and shCXCL12 transfected ECSCs were examined using western blot analysis. **D**. Samples were obtained from rhCXCL12 treated ECSCs respectively at 0, 5, 10 and 15 minutes for examination of ERK1/2 and p-ERK1/2 expression. **E**. The expression of ERK1/2 and p-ERK1/2 in vector and shCXCL12 transfected ECSCs were examined using western blot analysis. Statistically-significant differences were determined using an unpaired Student's t-test, *p<0.05, **p<0.01.

By the above studies, we demonstrated that ECSCs can maintain its characteristics of high-level invasion and metastasis by autocrine secretion of CXCL12 which binds to its receptor CXCR4, thereby plays an important role in the invasion and metastasis of esophageal cancer [12–14]. So how does CXCL12/CXCR4 maintain ECSCs’ characteristics of high-level invasion and metastasis? It has been reported that the CXCL12/CXCR4 axis can participate in various biological actions of tumors by activating the extracellular signal-regulating kinase 1/2 (ERK1/2) [15–18]. More importantly, it is strongly correlated with the participation of ERK1/2 pathway in tumor invasion and metastasis [19–20]. Based on this, we first compared the activation of the ERK1/2 pathway on ECSCs and non-ECSCs. The results showed that both OE33 cell derived ECSCs and EAC derived ECSCs had significantly stronger p-ERK1/2 activity compared with their corresponding non-ECSCs (Figure 5A, p<0.01), while no significant difference was seen in the expression of ERK1/2 (Figure 5A). We further examined the effect of the CXCL12-CXCR4 chemokine axis on the p-ERK1/2 pathway of ECSCs, and found that at 5 minutes after adding the CXCR4 inhibitor AMD-070, the activity of p-ERK1/2 on ECSCs was significantly inhibited while the level of ERK1/2 expression was not affected (Figure 5B, p<0.01, p<0.05). We also observed significant down-regulation of the p-ERK1/2 activity in shCXCR4 transfected ECSCs (Figure 5C, p<0.01). This indicated that the expression of CXCR4 was of crucial importance to the activation of the ERK1/2 signal pathway. Furthermore, we added rhCXCL12 to ECSCs and observed significant up-regulation of p-ERK1/2 (Figure 5D, p<0.01), while the activity of p-ERK1/2 was significantly reduced after ECSCs was transfected with shCXCL12 (Figure 5E, p<0.01). The above results sufficiently proved that the CXCL12-CXCR4 chemokine axis was of vital importance to the activation of ERK1/2 signal pathway on ECSCs.

### ECSCs’ strong disposition of invasion and metastasis depends on CXCL12/CXCR4 activated ERK1/2 signal pathway

**Figure 6:**
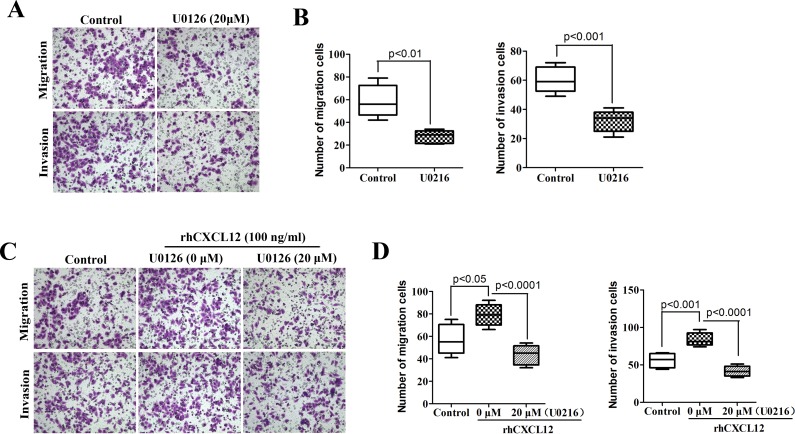
High propensity of invasion and metastasis of ECSCs depends on the CXCL12/CXCR4 activated ERK1/2 pathway **A**. ECSCs were added with ERK1/2 inhibitor U0126 (20μM) and incubated for 24 hours, the change in ECSCs’ disposition of invasion and metastasis was examined by transwell migration and matrigel invasion assays. **B**. After treatment with U0126, statistical analysis was done on ECSCs’ disposition of invasion by transwell migration and matrigel invasion assays. **C**. ECSCs were added with control, U0126 (20μM) and U0126 (20μM) & rhCXCL12 (100 ng/ml) for 24 hours, the change of invasion and metastasis was examined. **D**. Statistical analysis on migration and invasion array. Statistically-significant differences were determined using an unpaired Student's t-test.

The above results proved that CXCL12-CXCR4 chemokine axis is of vital importance to the activation of the ERK1/2 signal pathway on ECSCs. So is the ERK1/2 signal pathway essential to the enhancement of ECSCs’ disposition of invasion and metastasis by CXCL12/CXCR4? First, we treated ECSCs with an ERK1/2 signal pathway inhibitor U0126 (20μM) for 24 hours, and examined the change in ECSCs’ disposition of invasion and metastasis by transwell migration and matrigel invasion assays. The result showed that, just as blockage of the CXCL12/CXCR4 axis, blockage of the ERK1/2 signal pathway could result in significant inhibition of ECSCs’ disposition of migration and invasion (Figure 6A-6B, p<0.01, p<0.001). This suggested that the ERK1/2 signal pathway was of vital importance to the maintenance of ECSCs’ high disposition of migration and invasion. The above results confirmed that rhCXCL12 can significantly enhance ECSCs’ disposition of invasion and metastasis (Figure 4A-4B) and up-regulate the level of p-ERK1/2 (Figure 5D). Therefore, in addition to adding rhCXCL12 to the ECSCs migration and invasion models, we also added U0126 in order to block the ERK1/2 signal pathway, and the enhanced ability of ECSCs to invade and metastasize caused by rhCXCL12 was significantly rescued (Figure 6C-6D, p<0.05, p<0.01, p<0.001). The above results proved that the CXCL12/CXCR4 axis enhanced the strong ability of ECSCs to invade and metastasize.

## DISCUSSION

The important role of ECSCs in the strong disposition of tumor recurrence and metastasis has been proven in various tumor types [3–4]. For esophageal cancer, as early as 2003, Okumura demonstrated that a group of P75NTR-positive cells of normal esophageal epithelium were capable of proliferation, self-renewal and multidirectional differentiation, and were identified as esophageal epithelial stem cells [5]. Thereafter, numerous studies have been carried out on ECSCs. However, most of these studies focused on the molecular mechanism maintaining the self-renewal ability of ECSCs. For example, Zhang HF reported that PI3K/AKT/c-MYC axis played an important role in the maintenance of stemness of ECSCs [21]. Sato F et al. reported that EGFR inhibitors inhibited the formation of ECSCs by blocking the EMT transformation of epithelial cancer cells [6]. MiR-203 inhibited the self-renewal of ECSCs by targeting the stem cell self-renewal regulator Bim-1 [22]. Although Li S et al. reported that high expression of stem cell marker CD271 was closely associated with the patient's tolerability of chemotherapy and susceptibility to tumor invasion and metastasis [23], Forghanifard MM et al. found that the expression levels of stem cell regulators SALL4 and SOX2 in esophageal cancer tissues were closely correlated with poor prognosis and high metastasis rate in the patients [6]. The above study suggested that ECSCs play an important role in the invasion and metastasis of esophageal cancer, while its mechanism is still to be clarified.

Some studies found that in CXCR4-positive esophageal cancer, the level of CXCL12 expression was significantly correlated with lymphatic metastasis [12]; Koishi K et al. found that continuous up-regulation of CXCR4 in patients with esophageal cancer caused by radiotherapy and chemotherapy was significantly correlated with poor prognosis [13]; Numerous studies showed that radiotherapy and chemotherapy could gather or up-regulate the ratio of tumor stem cells, indirectly indicating that esophageal cancer might highly express CXCR4. More importantly, researcher examined 154 esophageal cancer tissue samples and found that simultaneous high expression of CD133 and CXCR4 was significantly correlated with shortened disease-free survival and overall survival in patients, suggesting that high expression of CXCR4 on CD133+ esophageal cancer stem cells was significantly correlated with poor prognosis [14]. The above report indirectly suggested that the CXCL12-CXCR4 chemokine axis is essential to the participation of ECSCs in the invasion and metastasis of esophageal cancer, while its specific effect and molecular mechanism are still to be clarified. This study found that ECSCs could autocrinely secrete large amount of chemokine CXCL12 and had high expression of its corresponding receptor CXCR4. More importantly, this study sufficiently proved the important role of the CXCL12-CXCR4 chemokine axis in the invasion and metastasis of ECSCs by loss-of-function and gain-of-function strategies and *in vivo* and *in vitro* experiments. The study provided new evidence on the participation of ECSCs in the invasion and metastasis of esophageal cancer and corresponding theoretical basis for clinical studies to prove the close correlation between the CXCL12-CXCR4 chemokine axis and poor prognosis and high recurrence and metastasis in the patients.

Some studies reported that the CXCL12-CXCR4 chemokine axis in brain glioma could activate extracellular signal regulating kinase 1/2 (ERK1/2) and AKT, degrade collagen fibers and induce proliferation of tumor cells [15]; the CXCL12-CXCR4 axis mediated chemotaxis and migration of T-cells by activating the MAPK kinase pathway ERK1/2 molecules [16]; A study on head and neck squamous cell carcinoma found that CXCL12 induced rapid mobilization of intracellular calcium ions, activation of ERK1/2, increase of MMP-9 secretion and degradation of basement membrane, ultimately enhancing the invasion and metastasis of cancer cells [17]. In a study on non-small cell lung cancer, researchers also found that the CXCL12-CXCR4 axis could induce the phosphorylation of ERK1/2, thereby enhancing proliferation of non-small cell lung cancer tumor cells [18]. More importantly, many reports indicated that the ERK1/2 pathway was closely correlated with tumor invasion and metastasis: p-ERK1/2 expression was closely correlated with metastasis of gastric adenocarcinoma, and enhanced p-ERK1/2 activity could significantly enhance the invasion and metastasis of gastric adenocarcinoma cells [19]. Our study found that ECSCs had increased p-ERK1/2 activity compared with normal esophageal cancer cells, and blockage of CXCL12 or CXCR4 could significantly inhibit the activity of p-ERK1/2, while adding rhCXCL12 could significantly enhance the activity of p-ERK1/2, therefore confirming that ECSCs maintained high activity of p-ERK1/2 by the CXCL12-CXCR4 chemokine axis. More importantly, when the ERK1/2 pathway was blocked by an inhibitor, the ability of ECSCs to invade and metastasize was significantly inhibited, and the up-regulation of ECSCs’ ability to migrate and invade could be reverted. Taken together, our findings suggested that autocrine CXCL12/CXCR4 was one of the major mechanisms underlying the metastatic property of ECSCs through ERK1/2 signaling pathway. This study filled the gap in the molecular mechanism by which ECSCs involve in invasion and metastasis, and provided a new molecular target for the prevention and treatment of esophageal cancer.

## MATERIALS AND METHODS

### Cell lines and cell culture

Human poorly differentiated EAC cell line OE33 cell was obtained from the European Collection of Cell Cultures (ECACC, Salisbury, UK). These cells were cultured in Dulbecco's modified Eagle's medium (DMEM) supplemented with 10% heat inactivated FBS (Hyclone), 100 units/ml penicillin and streptomycin. Esophageal carcinoma stem cell (ECSCs) were isolated from OE33 cell using Hoechst 33342 dye or CD133 markers [7], and cultured under stem cell conditions as previous described: 5μg/ml insulin, 0.4% bovine serum albumin, 20 ng/ml human recombinant epidermal growth factor, 10 ng/ml basic fibroblast growth factor, and were incubated in a 37°C incubator with 5% CO2. To establish stable low CXCL12 and CXCR4 expression ECSCs, ECSCs were transduced with lentivirus carrying CXCR4-shRNA or CXCL12-shRNA (GFP-shRNA as the control), as described previously. In migration and invasion experiment, ECSCs were treated with CXCR4 inhibitor (AMD-070, Sigma), rhCXCL16 (20 ng/ml, R&D Systems) or ERK1/2 inhibitor U0126 (20Mm, Sigma) for 24 hours *in vitro*. In other experiments, ECSCs were treated with CXCR4 inhibitor (AMD-070) or rhCXCL16 (20 ng/ml) for 5 min, 10 min or 15 min.

### Patient samples

Esophageal cancer tissues were collected by surgical resection at Daping Hospital (Chongqing, China) from Oct 2014 to Mar 2016. The patient details are shown in Supplementary Table 2.

### Matrigel invasion and transwell migration assays

In the transwell migration assay, 24-well culture inserts with porous polycarbonate membrane (8.0μm, Millipore) were used, and in the matrigel invasion assay, 30μl matrigel (BD Biosciences) was evenly smeared on the bottom of the upper chamber of transwell insert and incubated for 3 hours to be ready for use. Then 2 × 10^5^ cells were added into the upper chamber and 5% serum medium 800μl was added into the lower chamber. After conventional culture for 24 hours, the transwell insert was taken out. The endothelial cells and microglia on the chambers were wiped out with a cotton swab. 4% raformaldehyde was added for fixation. Crystal violet was added and the cells were incubated for 2 minutes for staining. Then PBS was added to turn the cells blue. The microscope was set at a magnification of 200x and 5 fields of view were chosen for which the mean value was calculated.

### RNA extraction and real-time PCR analysis

RNA extraction and real-time PCR were performed as described previously [7]. The primers for human CXCR4, CXCL12 and β-actin are listed in Supplementary Table 1. The qRT-PCR analysis was performed using the QuantiTect SYBR-Green PCR kit (Qiagen). Each experiment was performed at least three times. The relative expressions were calculated by normalization to β-actin gene expression.

### ELISA

OE33 cells and ECSCs and non-ECSCs obtained from tissues of patients with human esophageal cancer were seeded in 24-well plates at a concentration of 2×10^5^ cells/ml. After conventional culture for 48 hours, the supernatant was collected and frozen at -20°C for use. The concentration of CXCL16 protein was determined using an ELISA kit (R&D Systems) according to the manufacturer's protocol. The samples were measured with a spectrophotometer at a wavelength of 450 nm with triplicates for each sample.

### Flow cytometric analysis

In flow cytometry, cells were digested into single cell suspension. After washing with PBS, liquid FITC-conjugated CXCR4 antibody (BD Biosciences) was added and the suspension was incubated on ice for 30 minutes. After washing with PBS twice, CXCR4 expression was examined by a flow cytometry. Vector and shCXCR4 transfected esophageal cancer stem cells were digested into single cells, and were then washed twice and place on the machine for test.

### Immunofluorescence assay

In immunofluorescence assay, ECSCs were centrifuged and prepared into slides by a compact cytocentrifuge system. Cold 4% paraformaldehyde was added and the slides were allowed to fix for 10 minutes. BSA was added, and the slides were incubated at ambient temperature for 20 minutes. Mouse anti-human CXCR4 antibody (BD Bioscience) was added, and the slides were incubated overnight in the dark at 4°C. After washing with PBS for three times, Cy3-conjugated anti-mouse antibody (BD Bioscience) was added, and the slides were incubated at ambient temperature for 30 minutes. After washing with PBS, the nuclei were counterstained with DAPI.

### CCK8 array

The cells were digested into single cell suspension, and were seeded in a 96-well plate at a concentration of 5×10^5^ cells per well. A quadruplicate was used for each set of sample. After incubation for 0 hour, 24 hours and 48 hours, the samples were taken out. CCK8 reagent (Dojindo, 10μl/100μl culture system) was added into the plate 2 hours before the assay. 10μl 1%SDS was added to terminate the reaction. The samples were measured with a spectrophotometer at a wavelength of 450 nm with triplicates for each sample, and the mean values were calculated.

### Western blot

The expression of p-ERK1/2, ERK1/2, and α-tubulin by OE33 Cells or EAC derived ECSCs, or ECSCs transferred with shCXCL12 or shCXCR4, or treated with AMD-070 were determined by western blot analysis. The preparation of cell lysates and the Western blot analysis were performed as described previously [7]. Antibodies against p-ERK1/2 (1:200, Santa Cruz Biotechnology), ERK1/2 (1:200, Santa Cruz Biotechnology), and α-tubulin (1:500, Boster) were used. Each experiment was repeated for at least three times.

### *In vivo* experiments

Severe combined immunodeficient (SCID) mice were purchased from the Chinese Academy of Medical Sciences (Beijing, China). Mice were housed and maintained in laminar flow cabinets under specific pathogen free conditions. In the mouse subcutaneous tumor transplantation study, 1×10^6^ vector or shCXCR4 transfected esophageal cancer stem cells were implanted in the left thighs of the mice. Six mice per group. The volumes of the tumors were measured every three days, and statistical analysis was performed; at 50 days, the mice were euthanized, and the tumors were weighted. In the mouse tumor xenograft model, 1×10^6^ vector or shCXCR4 transfected esophageal cancer stem cells were injected into the bodies of the mice through the caudal vein. Six mice per group. At 60 days, the mice were euthanized, and pulmonary metastatic nodules were counted; the lung tissues were fixed with paraformaldehyde and HE staining was done.

### Statistical analysis

Independent sample t-test and one-way analysis of variance were carried out for all data using the SPSS17.0 software, and empirical data were presented by mean±SD. The difference was considered statistically significant when *p*<0.05. *p<0.05, **p<0.01, ***p<0.001.

## SUPPLEMENTARY MATERIALS FIGURES AND TABLES


