# Supplementary material for: Stem cell autocrine CXCL12/CXCR4 stimulates invasion and metastasis of esophageal cancer
Source: Oncotarget. 2017 Feb 10;8(22):36149–60. doi: 10.18632/oncotarget.15254 (PMC5482645; doi:10.18632/oncotarget.15254)
Supplement: Supplementary file 1 [file oncotarget-08-36149-s001.pdf]

Stem cell autocrine CXCL12/CXCR4 stimulates invasion and metastasis of esophageal cancer

SUPPLEMENTARY TABLES AND FIGURES

Supplementary Table 1: Primers for selected genes

Gene name	Primers		Product bp
	Sense	Antisense	
CXCR4	GGAGGGGATCAGTATATACA	GAAGATGATGGAGTAGATGG	145
CXCL12	GAGCCAACGTCAAGCATCTG	CGGGTCAATGCACACTTGTC	227
β-actin	TGGAGAAGAGCTATGAGCTGCCTG	GTGCCACCAGACAGCACTGTGTTG	201

Supplementary Table 2: Information about the esophageal cancer patients

ID	Sex	Age	Disease grade
01210262	F	44	Ia
01249260	F	23	Ia
05599012	F	42	Ia
03852213	F	36	Ib
04192631	M	46	Ib
04571312	F	56	Ib
04172547	F	44	IIa
02017530	M	31	IIa
04025749	M	54	IIa
02902032	F	60	IIb
04977667	M	55	IIIa
03034475	M	52	IIa
04094354	F	49	IIa
01220214	F	35	IIIa
03885259	M	47	IIa
02096126	M	68	IIa

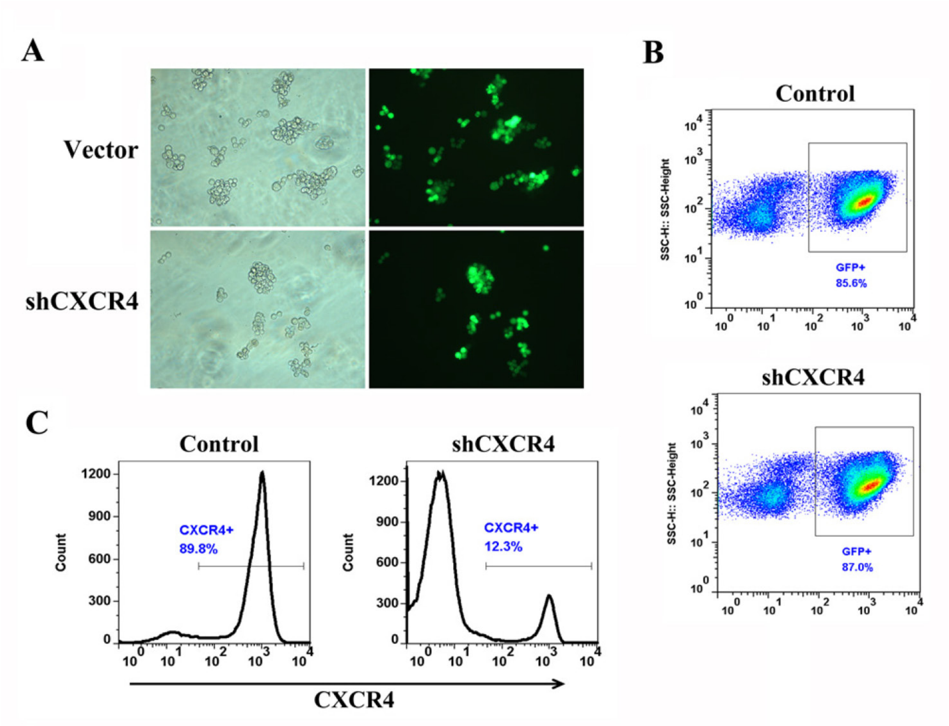

Supplementary Figure 1: A. A morphological image of ECSCs cells transfected with shCXCR4 or empty lentiviral vectors. **B.** Flow cytometric analysis of GFP expression in ECSCs transduced with vector or shCXCR4. **C.** Flow cytometric analysis of CXCR4 expression in ECSCs transduced with vector or shCXCR4.

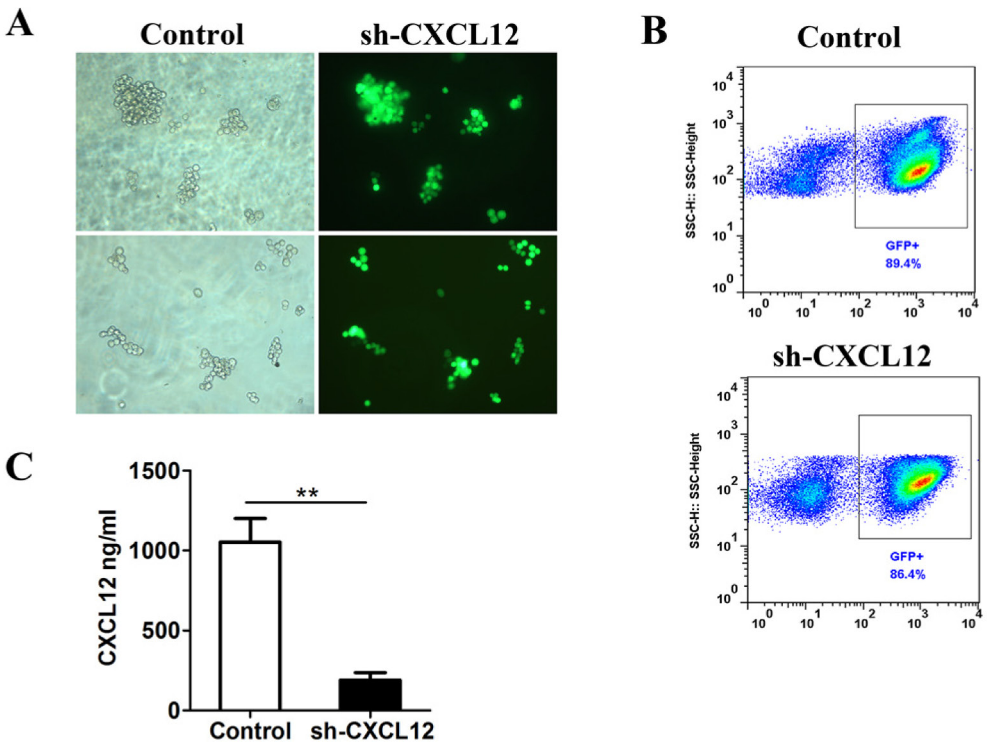

Supplementary Figure 2: A. A morphological image of ECSCs cells transfected with shCXL12 or empty lentiviral vectors. **B.** Flow cytometric analysis of GFP expression in ECSCs transduced with vector or shCXL12. **C.** ELISA analysis of CXCL12 expression in ECSCs transduced with vector or shCXL12. Statistically-significant differences were determined using an unpaired Student's t-test, $^{**}p<0.01$.
